# Results of Latissimus Dorsi Transfer Associated with the Use of Autologous Fascia Lata Tendon Graft in Irreparable Posterosuperior Rotator Cuff Injuries

**DOI:** 10.1055/s-0046-1819619

**Published:** 2026-04-22

**Authors:** Ricardo Makoto Okamoto, Guilherme do Val Sella, Luciana Andrade da Silva, Thomas Yogo Mendonça Alves, Alberto Naoki Miyazaki

**Affiliations:** 1 Shoulder Surgery Group, Department of Orthopedics and Traumatology, Faculdade de Ciências Médicas da Santa Casa de São Paulo, São Paulo, SP, Brazil

**Keywords:** rotator cuff, tendon transfer, transplantation, autologous, wounds and injuries, ferimentos e lesões, manguito rotador, transferência tendinosa, transplante autólogo

## Abstract

**Objective:**

To evaluate the functional outcomes of latissimus dorsi transfer reinforced with an autologous fascia lata graft harvested from the thigh contralateral to the injury to treat irreparable posterosuperior rotator cuff tears (IPSRCTs). The secondary objective was to assess complications related to graft harvesting.

**Methods:**

The present retrospective functional evaluation involved 17 patients (8 men and 9 women), with a mean age of 62.6 (54–73) years and a mean postoperative follow-up period of 21.2 (12–45) months. We compared pre- and postoperative University of California, Los Angeles (UCLA) scores and range of motion (ROM), assessed donor-site hip abduction strength and recorded potential associated complications. The determination of correlations between variables used Pearson's coefficient, with *p* ≤ 0.05 considered statistically significant.

**Results:**

The mean postoperative UCLA score was 27.9 (14–33) points, representing an improvement of 16.7 points compared to the preoperative mean. Range of motion improved significantly ( *p* < 0.05), with the following final values: elevation, 27.9°; lateral rotation (LR), 133°; medial rotation (MR) at T9, all with *p* < 0.05. No patient presented signs of infection at the graft harvest site; two subjects reported pain in the surgical wound, and one developed a small incisional hernia.

**Conclusion:**

This technique was effective in treating IPSRCTs, achieving a 70.6% satisfactory outcome based on the UCLA score and showing significant improvement in ROM. The use of an autologous graft represents a viable option with low complication rates, both at the shoulder and hip.

## Resumo

**Objetivo:**

Avaliar os resultados funcionais dos pacientes submetidos à transferência do latíssimo do dorso reforçado com um enxerto autólogo de fáscia lata retirado da coxa contralateral à lesão no tratamento das lesões posterossuperiores irreparáveis do manguito rotador (LPSIMRs). O objetivo secundário foi avaliar as complicações da retirada do enxerto.

**Métodos:**

Avaliação funcional retrospectiva de 17 pacientes (8 homens e 9 mulheres) com média de idade de 62,6 (54–73) anos e seguimento pós-operatório médio de 21,2 (12–45) meses, sendo comparados o University of California, Los Angeles (UCLA) e a amplitude de movimento (ADM) dos períodos pré e pós-operatório. Foram avaliados também se houve perda de força de abdução do quadril doador do enxerto e possíveis complicações associadas. As correlações entre as variáveis foram determinadas pelo coeficiente de Pearson, com valores de *p* ≤ 0,05.

**Resultados:**

A pontuação média UCLA do período pós-operatório foi de 27,9 (14–33) pontos com melhora de 16,7 pontos da média pré-operatória. Houve melhora das ADMs com os seguintes valores finais: elevação 27,9°; rotação lateral (RL) 133°; rotação medial (RM) em T9, todos com *p* < 0,05. Em relação ao local da retirada do enxerto, nenhum paciente apresentou sinais de infecção, dois se queixaram de dor na ferida operatória e um apresentou pequena hérnia incisional.

**Conclusão:**

A técnica realizada foi eficaz no tratamento de LPSIMRs, com 70,6% de resultados satisfatórios de acordo com a pontuação UCLA e melhora da ADM. O uso do enxerto autólogo é uma opção viável com baixos índices de complicações, tanto no ombro quanto no quadril.

## Introduction

Rotator cuff tears may cause severe functional impairment and disabling shoulder pain in affected subjects, often requiring surgical treatment. 1 Among these injuries, irreparable rotator cuff tears (IRCTs) represent a major challenge for shoulder surgeons. Several surgical techniques aim to improve pain and shoulder function, including reverse arthroplasty, 2 superior capsule reconstruction, 3 4 and tendon transfers, such as trapezius tendon transfer 5 or latissimus dorsi tendon (LDT) transfer. 6 7 8 9 10 11 12 13 These structures may incorporate reinforcement with homologous or autologous tendon grafts. Pogorzelski et al. 14 used a homologous Achilles tendon graft to reinforce LDT transfers in 16 patients with IRCT. In a systematic review, Bouchard et al. 15 evaluated the outcomes and complications of tendon transfers for IRCT repair and reported that LDT transfer was the most performed technique.

In 1988, Gerber et al. 6 described LDT transfer to the greater tuberosity through a dual (superolateral and posterior) approach for rotator cuff tears in which direct repair at the native attachment site was not feasible even after tendon mobilization and release. 1 Some authors reported favorable outcomes in pain relief and functional improvement using this technique. 7 12 However, failure rates reached 36% in patients treated according to Gerber's technique. 6 13 Evidence indicates that most failures occurred for two primary reasons. The first is the detachment of the transferred tendon from its reattachment site, 13 and the second refers to the deltoid origin dehiscence due to transfer tension and the thickness of the LDT. 16 In 2019, Miyazaki et al. 11 described a modified LDT transfer technique incorporating elongation with a homologous graft for IRCT treatment, modifying the original method proposed by Gerber et al. 6 to prevent both complications simultaneously. Their modifications included elongating and reinforcing the LDT with a homologous tendon graft obtained from a tissue bank and performing the transfer through a single deltopectoral approach. 11 Checchia et al. 17 demonstrated that mid-term functional outcomes were superior with the modified technique described by Miyazaki et al. 18 compared with the original technique from Gerber et al. 6

However, homologous graft use remains limited by cost and donor scarcity. Imai et al. 19 used an autologous tensor fasciae latae (TFL) graft in 39 patients with IRCT associated with pseudoparalysis, reporting that this graft type represents a viable option. Similarly, Pochini et al. 20 used an autologous TFL graft harvested by a minimally-invasive technique to reinforce pectoralis major reconstruction, further supporting the feasibility of this approach in tendon reconstructions.

The primary objective of the current study is to evaluate the functional outcomes of elongated LDT transfers reinforced with autologous TFL grafts for the treatment of IPSRCTs. The secondary objective is to assess complications related to graft harvesting from the thigh.

## Methods

The present retrospective study evaluated the clinical and epidemiological data of 21 patients with IRCTs who underwent treatment using an LDT transfer, elongated with an autologous TFL tendon graft harvested from the thigh contralateral to the affected shoulder. The procedure aimed to treat IPSRCTs. Surgeries occurred from November 2020 to July 2023. As two patients declined participation and two did not complete the minimum postoperative rehabilitation period, the final sample comprised 17 patients. The diagnosis was made based on a clinical examination and magnetic resonance imaging (MRI) and further confirmed during surgery.

Inclusion criteria comprised patients over 18 years old with IRCTs who underwent the specified technique, adhered to the rehabilitation protocol, and completed at least 1 year of follow-up. Exclusion criteria included refusal to participate and nonadherence to the rehabilitation protocol.

Of the 17 operated patients, 8 (47%) were male, and 9 (53%) female, with a mean age at the time of surgery of 62.6 (54–73) years. In total, 13 (76.5%) procedures involved the dominant shoulder, and the etiology was traumatic in 12 (70.6%) patients ( Table 1 ).

**Table 1:** Demographic and preoperative data

Case number	Gender	Age (years)	Dominance	Traumatic etiology	Symptoms (months)	Supra	Infra	Hamada et al. 21 classification	Follow-up (months)
1	F	64	+		24	4	2	3	45
2	F	67	+		120	3	3	2	33
3	F	57	+	+	4	3	3	2	28
4	M	66		+	20	4	3	1	26
5	M	62	+	+	5	2	3	1	25
6	M	70	+	+	12	2	2	2	25
7	F	55	+	+	7	4	2	3	23
8	F	73	+	+	72	3	3	1	19
9	M	54			72	4	4	4A	17
10	F	54		+	10	4	4	1	17
11	M	57	+	+	20	4	4	3	16
12	M	61		+	2	4	2	3	16
13	M	65	+	+	6	3	1	1	15
14	F	58	+	+	120	4	2	3	15
15	F	70	+		24	4	4	4A	15
16	M	65	+		12	3	4	1	13
17	F	66	+	+	4	3	2	2	12
Mean		62.6			31.4				21.2

**Abbreviations** : F, Female; infra, infraspinatus muscle; M, male; supra, supraspinatus muscle.

The mean interval between symptom onset and surgery was of 31.4 (2–120) months, and the mean postoperative follow-up was of 21.2 (12–45) months ( Table 1 ).

All patients underwent radiographic evaluation of the affected shoulder to assess signs of rotator cuff arthropathy, classified according to Hamada et al. 21 In addition, all subjects underwent an MRI scan, which assessed not only the tear but also fatty infiltration of the supraspinatus and infraspinatus muscles, classified according to the method described by Goutallier and modified by Fuchs et al. 22 ( Table 1 ).

According to the classification by Hamada et al., ^21^ 6 patients were categorized as grade 1, 4 as grade 2, 5 as grade 3, and 2 as grade 4A ( Table 1 ).

Based on the Goutallier classification modified by Fuchs et al., 22 supraspinatus involvement was grade II in 2 cases, grade III in 6, and grade IV in 9 cases. Infraspinatus involvement was grade I in 1 case, grade II in 6, grade III in 5, and grade IV in 5 cases ( Table 1 ).

**Fig. 1:**
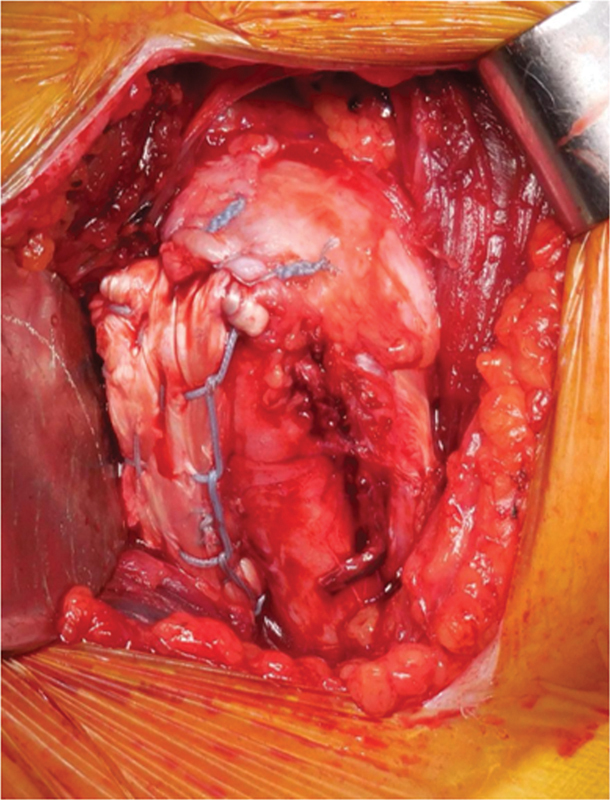
Lengthened latissimus dorsi transfer with autologous fascia lata graft fixed to the posterosuperior region of the greater tuberosity.

The LDT transfer technique and postoperative rehabilitation protocol followed the method described by Checchia et al., 13 except that we used an autologous TFL graft for fixation at the posterosuperior region of the greater tuberosity ( Fig. 1 ).

**Fig. 2:**
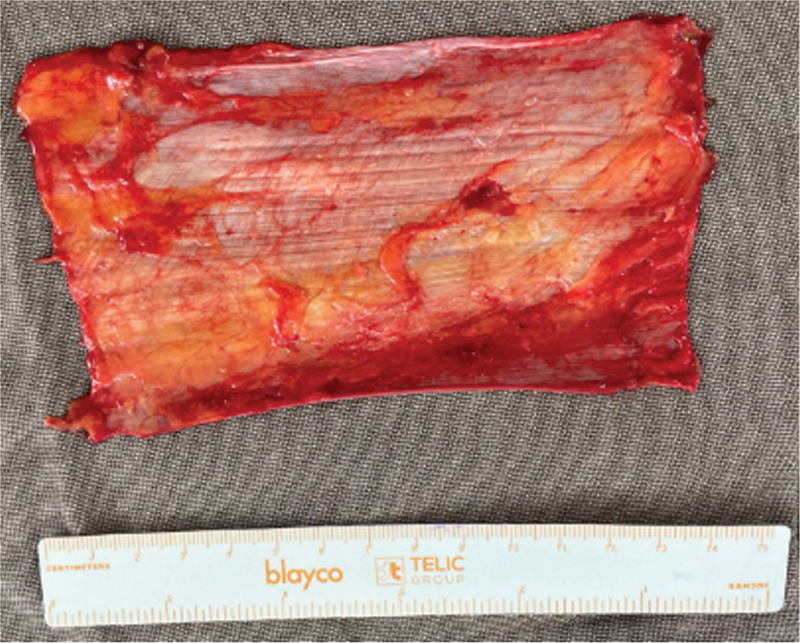
Autologous fascia lata graft measuring 15 cm in length and 5 cm in width, harvested from the anterolateral aspect of the thigh.

**Fig. 3:**
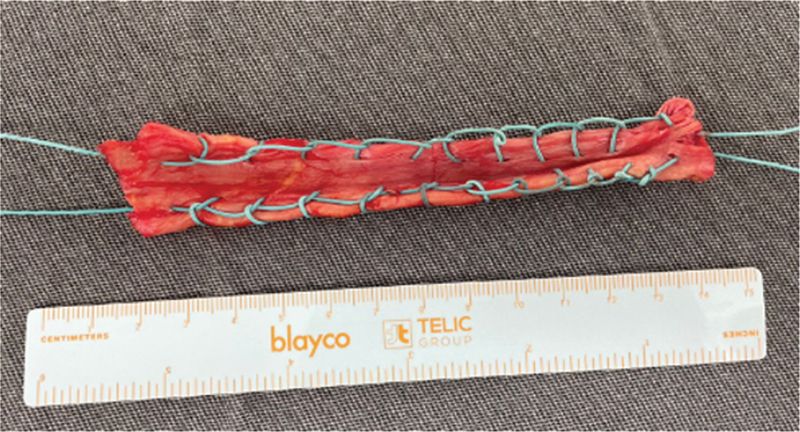
Autologous fascia lata graft prepared by folding it in half and suturing it with # 2 polyester threads.

**Fig. 4:**
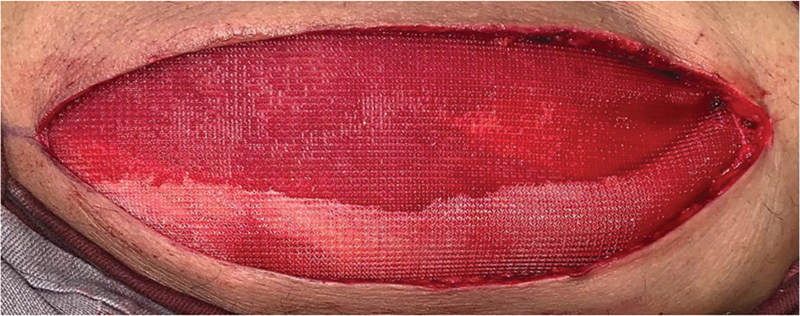
Closure of the surgical wound of the thigh contralateral to the rotator cuff lesion using polypropylene mesh.

We harvested the autologous TFL graft from the anterolateral aspect of the thigh contralateral to the affected shoulder through an incision approximately 15 cm in length. The graft measured 15 cm in length and 5 cm in width ( Fig. 2 ). We prepared it by folding it in half and reinforcing it with # 2 polyester sutures ( Fig. 3 ). After graft harvesting, the donor site was closed using a polypropylene surgical mesh ( Fig. 4 ).

We evaluated the donor site by comparing hip abduction strength with that of the contralateral side and assessing potential complications, including infection, local pain, incisional hernia, and adduction contracture using the Ober test. We measured hip abduction strength bilaterally with a properly calibrated dynamometer and compared values to determine whether strength loss occurred in the donor limb.

We recorded active pre- and postoperative ROM according to the method proposed by the American Academy of Orthopedic Surgeons. 23 Functional outcomes assessments used the University of California, Los Angeles (UCLA) score developed by Ellman et al. 24 We statistically compared pre- and postoperative ROM and functional scores.

Comparisons used paired t-tests for continuous variables within the same group and unpaired t-tests for continuous variables between different groups. Pearson's coefficient was employed to determine correlations between continuous variables. A *p* -value ≤ 0.05 indicated statistical significance.

The Institutional Research Ethics Committee approved the study under CAAE 79565024.6.0000.5479.

## Results

Mean active elevation improved from 96.5° preoperatively to 133° postoperatively ( *p* < 0.05). Mean active external rotation (ER) increased from 41.2° preoperatively to 52.8° postoperatively ( *p* < 0.05). Mean active internal rotation (IR) improved from the L2 level preoperatively to the T9 level postoperatively ( *p* < 0.05) ( Table 2 ).

**Table 2:** Assessment of range of motion and functional outcomes in the pre- and postoperative periods

Case number	ROM						UCLA	
	PRE ELEV (°)	POST ELEV (°)	PRE LR (°)	POST LR (°)	PRE MR	POST MR	PRE TOTAL	POST TOTAL
1	60	160	40	60	L3	T12	8	28
2	140	100	70	60	L2	T7	13	24
3	80	160	45	60	T7	T6	7	33
4	60	100	10	40	L4	T12	15	23
5	80	160	60	60	L2	T8	10	33
6	60	100	60	60	Gluteus	L5	27	14
7	80	100	60	90	L5	T7	6	30
8	50	100	50	60	L4	T5	13	33
9	150	160	45	45	T7	T12	12	31
10	90	100	30	30	L4	T7	5	21
11	90	160	80	90	Gluteus	T5	11	33
12	80	160	10	30	T7	T5	15	28
13	140	160	30	80	L4	T7	13	33
14	100	120	20	45	L5	L2	8	19
15	140	160	30	30	T8	T8	7	32
16	150	160	30	45	T7	T5	13	31
17	90	100	30	30	Gluteus	Gluteus	8	28
Mean	96.47	132.94	41.17	52.82	L2	T9	11.23	27.88
	*p* < 0.05		*p* < 0.05		*p* < 0.05		*p* < 0.05	

**Abbreviations** : ELEV, Elevation; LR, lateral rotation; MR, medial rotation; POST, postoperative; PRE, preoperative; ROM, range of motion.

The mean preoperative UCLA score was of 11.2 (7–27) points, whereas the mean postoperative score increased to 27.9 (14–33) points ( *p* < 0.05) ( Table 2 ). According to postoperative UCLA scores, 12 patients achieved satisfactory results (≥ 28 points) and 5 had unsatisfactory results (≤ 27 points). Three patients reported persistent pain after the procedure. In one case (case number 6), the UCLA score worsened due to rotator cuff retear.

Regarding the donor site, analysis of mean hip abduction strength (HAS) showed that 9 patients (53%) had lower HAS on the graft-harvested side compared with the contralateral side, 6 patients (35.3%) had higher HAS on the graft side, and 2 patients (11.76%) had equal strength bilaterally. However, comparison of mean HAS between the donor and contralateral limbs revealed no statistically significant difference ( *p* = 0.578).

No patient developed signs of infection at the TFL graft harvest site. Two patients reported pain at the surgical wound, and one developed an incisional hernia. We also assessed adduction contracture of the donor limb using the Ober test, and no patient had a positive result.

## Discussion

In the current study, 70.6% of patients achieved satisfactory clinical outcomes, supporting this technique as a potential alternative to reverse arthroplasty in patients with IRCTs. Miyazaki et al. 18 and Gerber et al. 1 reported similar results, observing satisfactory outcomes in 71% and 62% of their patients, respectively.

Active ROM improved significantly between the pre- and postoperative periods, with gains of 36.5° in elevation ( *p* < 0.05), 12.7° in LR ( *p* < 0.05), and 5 vertebral levels in MR ( *p* < 0.05) ( Table 2 ). These findings are consistent with those of Boileau et al., 9 who performed LDT transfer for IRCTs treatment in 15 patients and reported a 34.7° increase in elevation after surgery. Miyazaki et al. 18 used a homologous graft to reinforce LDT transfer, resulting in improvements in both ROM and UCLA scores as functional outcomes, like those observed in the present study.

Aoki et al. 7 performed 12 LDT transfers with a mean follow-up of 35.6 months and reported a 36° gain in elevation. Functional assessment using the UCLA score showed 75% satisfactory outcomes, with a mean score of 28 points. In contrast, Miniaci et al. 8 reported that in 17 patients undergoing LDT transfer with a mean postoperative follow-up of 51 months, the mean UCLA score improvement was modest, from 6.8 preoperatively to 16.4 postoperatively. In a systematic review, Bouchard et al. 15 evaluated 980 patients undergoing tendon transfers (mean age: 58.9 years; mean follow-up: 44.7 months). These authors noted that LDT transfer was the most used technique for IRCTs and reported a mean increase of 18.7 points in the UCLA score after surgery.

Several authors have used acellular dermal allografts for IRCT repair. 25 26 Gupta et al. 25 used an allograft to bridge the defect between the rotator cuff and its footprint in 24 patients (mean age: 63 years), reporting improved flexion and LR without graft-related complications. Skedros et al. 26 described a clinical case of elongated latissimus dorsi transfer reinforced with an acellular dermal allograft, achieving satisfactory outcomes with 180° of active flexion and 60° of LR.

Like our group, other authors have used autologous TFL 19 27 and iliotibial band (ITB) grafts 28 in IRCT repair. Mori et al. 27 compared arthroscopic autologous TFL graft reconstruction with arthroscopic partial repair in 48 patients with IRCTs (24 subjects per group). After an average follow-up period of 35 months, the graft group demonstrated better functional outcomes and a significantly lower retear rate of 8.3%, compared to the partial repair group, which had a retear rate of 41.7%. 27 Mihara et al. 28 observed that the use of ITB with a bone plug for IRCT repair resulted in no cases of graft retear during 2 years of follow-up. Imai et al. 19 used an autologous TFL graft in 39 patients younger than 60 years with IRCTs associated with pseudoparalysis and reported improvements in elevation (from 57–131°) and in LR (from 17–32°). As such, their study supports this approach as a viable option for younger patients with pseudoparalysis. 19

In a systematic review, Lewington et al. 29 compared 15 studies evaluating different graft types for IRCT repair and concluded that functional outcomes were comparable across graft types. However, autologous graft use was associated with higher donor-site morbidity and

longer operative time. In the present study, TFL graft harvesting did not result in clinically relevant donor-site complications nor prolonged operative time, as two surgical teams worked simultaneously—one performing shoulder exposure while the other harvested the graft. Furthermore, comparison of hip strength between the donor and contralateral limbs revealed no loss of abduction strength. Homologous tendon grafts may represent a long-term disadvantage due to concerns regarding graft survival. Evidence suggests higher failure rates in homologous grafts compared with autologous grafts. 30 Getgood et al. 30 compared autologous and homologous grafts in anterior cruciate ligament reconstruction and found a threefold higher rate of retear in patients treated with homologous grafts.

One more limitation of using homologous grafts is the regulatory and logistical requirements involved. In Brazil, surgeons and hospitals must register with the Ministry of Health before obtaining authorization to use these grafts. Even after registering, the availability of donors remains limited due to a shortage in tissue banks. Given these constraints—limited availability, regulatory requirements, and procurement logistics—autologous grafting becomes a practical and reliable alternative with low complication rates.

The current study has limitations. We did not objectively compare pre- and postoperative strength, as functional assessment relied solely on the UCLA score. In addition, postoperative follow-up was relatively short; previous studies in the literature have reported minimum follow-up periods of 24 months.

## Conclusion

In the present study, after a minimum follow-up of 12 months, the technique used for the treatment of IPSRCTs resulted in 70.6% satisfactory outcomes according to the UCLA score, along with improvement in elevation, LR, and MR. The use of an autologous graft is a viable option, with a complication rate of 17.6% in this sample, observed both at the shoulder and the graft donor site.

## References

[1] Gerber C Maquieira G Espinosa N Latissimus dorsi transfer for the treatment of irreparable rotator cuff tears J Bone Joint Surg Am 2006 88 01 113 120 10.2106/JBJS.E.00282 16391256

[2] Mulieri P Dunning P Klein S Pupello D Frankle M Reverse shoulder arthroplasty for the treatment of irreparable rotator cuff tear without glenohumeral arthritis J Bone Joint Surg Am 2010 92 15 2544 2556 10.2106/JBJS.I.00912 21048173

[3] Mihata T Lee T Q Watanabe C Clinical results of arthroscopic superior capsule reconstruction for irreparable rotator cuff tears Arthroscopy 2013 29 03 459 470 10.1016/j.arthro.2012.10.022 23369443

[4] Gracitelli M EC Beraldo R A Malavolta E A Assunção J H Oliveira DRO Ferreira Neto A A Superior Capsular Reconstruction with Fascia Lata Allograft for Irreparable Supraspinatus Tendon Tears Rev Bras Ortop 2019 54 05 591 596 10.1016/j.rbo.2017.11.011 PMC6819162 31686714

[5] Elhassan B T Wagner E R Werthel J-D Outcome of lower trapezius transfer to reconstruct massive irreparable posterior-superior rotator cuff tear J Shoulder Elbow Surg 2016 25 08 1346 1353 10.1016/j.jse.2015.12.006 26968088

[6] Gerber C Vinh T S Hertel R Hess C W Latissimus dorsi transfer for the treatment of massive tears of the rotator cuff. A preliminary report Clin Orthop Relat Res 1988 232 51 61 3383502

[7] Aoki M Okamura K Fukushima S Takahashi T Ogino T Transfer of latissimus dorsi for irreparable rotator-cuff tears J Bone Joint Surg Br 1996 78 05 761 766 8836066

[8] Miniaci A MacLeod M Transfer of the latissimus dorsi muscle after failed repair of a massive tear of the rotator cuff. A two to five-year review J Bone Joint Surg Am 1999 81 08 1120 1127 10.2106/00004623-199908000-00007 10466644

[9] Boileau P Chuinard C Roussanne Y Neyton L Trojani C Modified latissimus dorsi and teres major transfer through a single delto-pectoral approach for external rotation deficit of the shoulder: as an isolated procedure or with a reverse arthroplasty J Shoulder Elbow Surg 2007 16 06 671 682 10.1016/j.jse.2007.02.12 18061113

[10] Merolla G Chillemi C Franceschini V Tendon transfer for irreparable rotator cuff tears: indications and surgical rationale Muscles Ligaments Tendons J 2015 4 04 425 432 25767779 PMC4327351

[11] Miyazaki A N Checchia C S de Castro Lopes W Fonseca Filho J M Sella GDV da Silva LA Latissimus Dorsi Tendon Transfer using Tendinous Allograft for Irreparable Rotator Cuff Lesions: Surgical Technique Rev Bras Ortop 2019 54 01 99 103 10.1055/s-0038-1676989 PMC6415531 31363253

[12] Kany J Sekaran P Grimberg J Risk of latissimus dorsi tendon rupture after arthroscopic transfer for posterior superior rotator cuff tear: a comparative analysis of 3 humeral head fixation techniques J Shoulder Elbow Surg 2020 29 02 282 290 10.1016/j.jse.2019.06.019 31473133

[13] Checchia C S Silva LAD Sella GDV Fregoneze M Miyazaki A N Current Options in Tendon Transfers for Irreparable Posterosuperior Rotator Cuff Tears Rev Bras Ortop 2021 56 03 281 290 10.1055/s-0040-1709988 PMC8249074 34239191

[14] Pogorzelski J Horan M P Godin J A Hussain Z B Fritz E M Millett P J Achilles tendon allograft-augmented latissimus dorsi tendon transfer for the treatment of massive irreparable posterosuperior rotator cuff tears Arch Orthop Trauma Surg 2018 138 09 1207 1212 10.1007/s00402-018-2943-8 29876638

[15] Bouchard M D Patel N A Keogh C Evaluating tendon transfers in irreparable rotator cuff tears: A systematic review of clinical outcomes and failure rates Shoulder Elbow 2025 17585732251368884 17585732251368884 10.1177/17585732251368884 PMC12354401 40821879

[16] Sher J S Iannotti J P Warner J J Groff Y Williams G R Surgical treatment of postoperative deltoid origin disruption Clin Orthop Relat Res 1997 343 93 98 9345213

[17] Checchia C S da Silva LA do Val Sella G Checchia S L de Moraes Barros Fucs PM Miyazaki A N Allograft-enhanced latissimus dorsi transfer is better than the conventional technique for irreparable posterosuperior rotator cuff tears. A retrospective matched cohort Int Orthop 2023 47 06 1527 1534 10.1007/s00264-023-05775-0 36951977

[18] Miyazaki A N Checchia C S Fonseca Filho J M Rosa J RP Val Sella GD Silva LAD Results of Latissimus Dorsi Transfer using a Tendinous Allograft through a Single Deltopectoral Approach for Irreparable Posterosuperior Rotator Cuff Tears Rev Bras Ortop 2021 57 04 590 598 10.1055/s-0041-1724073 PMC9365491 35966441

[19] Imai S Graft-Augmented Repair of Irreparable Massive Rotator Cuff Tears with Latissimus Dorsi Transfer to Treat Pseudoparesis JB JS Open Access 2021 6 04 e21.00044 10.2106/JBJS.OA.21.00044 PMC8631387 34859173

[20] Pochini AdC Ejnisman B Andreoli C V Rupture of the bilateral and simultaneous tendon of the pectoralis major muscle. Description of three cases J Surg Case Rep 2023 2023 11 rjad531 10.1093/jscr/rjad531 38223468 PMC10641291

[21] Hamada K Fukuda H Mikasa M Kobayashi Y Roentgenographic findings in massive rotator cuff tears. A long-term observation Clin Orthop Relat Res 1990 254 92 96 2323152

[22] Fuchs B Weishaupt D Zanetti M Hodler J Gerber C Fatty degeneration of the muscles of the rotator cuff: assessment by computed tomography versus magnetic resonance imaging J Shoulder Elbow Surg 1999 8 06 599 605 10.1016/s1058-2746(99)90097-6 10633896

[23] American Academy of Orthopaedic Surgeons Joint Motion: Method of Measurement and Recording Edinburgh British Orthopaedic ASSN 1965

[24] Ellman H Hanker G Bayer M Repair of the rotator cuff. End-result study of factors influencing reconstruction J Bone Joint Surg Am 1986 68 08 1136 1144 3771595

[25] Gupta A K Hug K Berkoff D J Dermal tissue allograft for the repair of massive irreparable rotator cuff tears Am J Sports Med 2012 40 01 141 147 10.1177/0363546511422795 22215726

[26] Skedros J G Henrie T R Latissimus Dorsi Tendon Transfer with GraftJacket® Augmentation to Increase Tendon Length for an Irreparable Rotator Cuff Tear Case Rep Orthop 2017 2017 8.086065E6 10.1155/2017/8086065 PMC5282417 28194290

[27] Mori D Funakoshi N Yamashita F Arthroscopic surgery of irreparable large or massive rotator cuff tears with low-grade fatty degeneration of the infraspinatus: patch autograft procedure versus partial repair procedure Arthroscopy 2013 29 12 1911 1921 10.1016/j.arthro.2013.08.032 24169146

[28] Mihara S Fujita T Ono T Inoue H Kisimoto T Rotator cuff repair using an original iliotibial ligament with a bone block patch: preliminary results with a 24-month follow-up period J Shoulder Elbow Surg 2016 25 07 1155 1162 10.1016/j.jse.2015.11.015 26899035

[29] Lewington M R Ferguson D P Smith T D Burks R Coady C Wong I H-B Graft Utilization in the Bridging Reconstruction of Irreparable Rotator Cuff Tears: A Systematic Review Am J Sports Med 2017 45 13 3149 3157 10.1177/0363546517694355 28345960

[30] Getgood A Hamstring Autograft Had Better Long-Term Survivorship Than Tibialis Posterior Tendon Allograft for Anterior Cruciate Ligament Reconstruction J Bone Joint Surg Am 2016 98 10 872 10.2106/JBJS.16.00209 27194499

